# Recombinant Live Attenuated Influenza Virus Expressing Conserved G-Protein Domain in a Chimeric Hemagglutinin Molecule Induces G-Specific Antibodies and Confers Protection against Respiratory Syncytial Virus

**DOI:** 10.3390/vaccines8040716

**Published:** 2020-12-01

**Authors:** Yu-Jin Jung, Yu-Na Lee, Ki-Hye Kim, Youri Lee, Subbiah Jeeva, Bo Ryoung Park, Sang-Moo Kang

**Affiliations:** 1 Center for Inflammation, Immunity & Infection, Institute for Biomedical Sciences, Georgia State University, Atlanta, GA 30303, USA; pharmaco12@gmail.com (Y.-J.J.); mistybluerain7@gmail.com (Y.-N.L.); kkim39@gsu.edu (K.-H.K.); youl6248@gmail.com (Y.L.); jsubbiah@gsu.edu (S.J.); bpark9@student.gsu.edu (B.R.P.); 2 Animal and Plant Quarantine Agency, Gimcheon, Gyeongsangbukdo 39660, Korea

**Keywords:** recombinant LAIV. RSV G, chimeric hemagglutinin

## Abstract

Respiratory syncytial virus (RSV) is one of the most important pathogens causing significant morbidity and mortality in infants and the elderly. Live attenuated influenza vaccine (LAIV) is a licensed vaccine platform in humans and it is known to induce broader immune responses. RSV G attachment proteins mediate virus binding to the target cells and they contain a conserved central domain with neutralizing epitopes. Here, we generated recombinant LAIV based on the attenuated A/Puerto Rico/8/1934 virus backbone, expressing an RSV conserved G-domain in a chimeric hemagglutinin (HA) fusion molecule (HA-G). The attenuated phenotypes of chimeric HA-G LAIV were evident by restricted replication in the upper respiratory tract and low temperature growth characteristics. The immunization of mice with chimeric HA-G LAIV induced significant increases in G-protein specific IgG2a (T helper type 1) and IgG antibody-secreting cell responses in lung, bronchioalveolar fluid, bone marrow, and spleens after RSV challenge. Vaccine-enhanced disease that is typically caused by inactivated-RSV vaccination was not observed in chimeric HA-G LAIV as analyzed by lung histopathology. These results in this study suggest a new approach of developing an RSV vaccine candidate while using recombinant LAIV, potentially conferring protection against influenza virus and RSV.

## 1. Introduction

Respiratory syncytial virus (RSV) is a non-segmented, negative-sense, and single-stranded RNA virus, which encodes 11 proteins. Annually, about two-million of children (<5 years old) are affected by RSV associated lower respiratory infection in the United States [1,2]. The hospitalization rate of the elderly (>65 years old) has increased during the last decades [2,3]. The development of a safe and effective RSV vaccine has proven to be difficult, since vaccine-enhanced respiratory disease was observed during epidemic season after formalin-inactivated RSV (FI-RSV) vaccination in young naïve children [4]. Several RSV vaccines are under clinical trials, but none has been licensed on the market [3,5].

RSV main surface glycoproteins, the F- and G-proteins, play important roles in inducing the host immune responses to RSV infection [5,6,7]. RSV G-protein comprises two conserved regions, a cytoplasmic/transmembrane region (aa 1–63) and a central conserved region (aa131–230). Among the different RSV strains circulating, there is a highly conserved region (aa 164–176) within the central G region, including B cell epitopes (aa 174–187) and T cell epitopes (aa 184–198) [5,8]. In addition, the CX3C chemokine motif (aa 182–186) in RSV G binds to CX3C receptor CX3CR1 mimicking several activities. The treatment of anti-G monoclonal antibody (mAb) 131-2G after RSV infection blocks G-protein binding to CX3CR1 in a mouse model [5,9,10,11].

Live attenuated influenza vaccine (LAIV) is a licensed influenza vaccine platform and it has been used since 2003 in the United States and 2012 in Europe [12]. LAIV is produced using six gene segments from the cold adapted master donor virus and hemagglutinin (HA) and neuraminidase (NA) genes encoding surface glycoproteins of vaccine strain by reverse genetics. The replication of LAIV is restricted in the upper respiratory tracts at lower temperature (32–34 °C) [13,14]. The high efficacy of LAIV was observed in young children and patients with asthma or human immunodeficiency virus [12]. Intranasal administration of LAIV induces mucosal immunity at the site of immunization. Furthermore, LAIV provokes increased memory B cells and T cells for more than six months in immunized children [15].

Recombinant influenza viruses containing RSV G- and F-protective epitopes have been reported. A neutralizing epitope fragment of RSV F-protein was inserted into HA in a chimeric conjugate, and expressed in the replication competent recombinant A/Puerto Rico/8/1934 (A/PR8) with pathogenic WT backbone, which was shown to provide protection against RSV in mice [16]. Similarly, recombinant WT A/PR8 carrying the RSV G-conserved domain was demonstrated to confer protection against RSV infection [17]. Recombinant A/PR8 virus with WT backbone is pathogenic in mice and it would not mimic the LAIV phenotypes as a viral vaccine vector.

In this study, we generated an A/PR8 backbone with attenuated phenotypes and a recombinant A/PR8 containing an RSV G-conserved domain in a chimeric HA conjugate of the attenuated A/PR8 backbone by reverse genetics. Recombinant attenuated A/PR8 with chimeric HA-G could induce IgG2a isotype dominant responses to an RSV-G foreign antigen and provide protection against RSV challenge in mice. The significance and limitations in developing recombinant influenza virus vectors expressing RSV neutralizing domains have been discussed.

## 2. Materials and Methods

### 2.1. Cells and Virus Preparation

The RSV strain A2 kindly provided by Dr. Martin Moore (Emory University) was amplified in Hep2 cells that were obtained from ATCC using Dulbecco’s modified eagle media including 10% fetal bovine serum and 1% penicillin/streptomycin antibiotics, as described [17,18]. Influenza virus A/Puerto Rico/8/34 (A/PR8, H1N1) was amplified in 10 to 11-day-old embryonated chicken eggs and stored at −80 °C until use. For the preparation of FI-RSV, RSV were grown in Hep2 cells and culture supernatants with cell debris centrifuged followed by filtration. The filtered RSV were then inactivated in formalin (1:4000 vol/vol) for 72 h at 37 °C and then adsorbed to aluminum hydroxide (4 mg/mL) to formulate for FI-RSV alum adjuvant as described [19].

### 2.2. Construction of LAIV A/PR8 Backbone and Recombinant Attenuated A/PR8 Virus Expressing Chimeric HA-G Proteins

**Figure 1:**
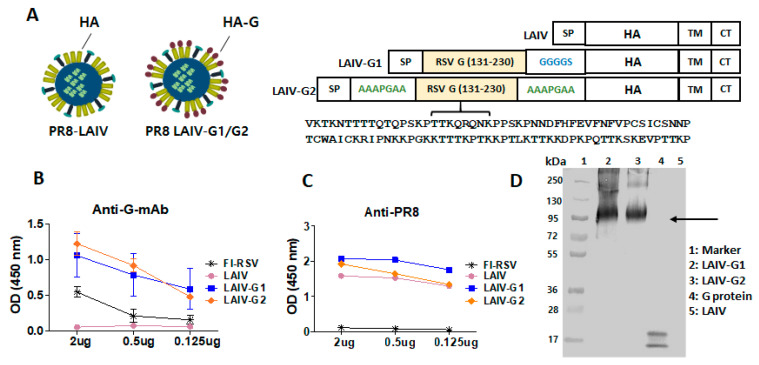
The characterization of recombinant live attenuated influenza vaccine (LAIV)-G1 and LAIV-G2 viruses. (**A**) Schematic representation of LAIV and recombinant LAIV expressing respiratory syncytial virus (RSV) G conserved glycoprotein aa131–130 domain with amino acid sequence indicated using different linkers. (**B**) RSV G specific mAb 131-2G reactivity of LAIV, LAIV-G1, and LAIV-G2 viruses. (**C**) Anti-A/PR8 serum reactivity of LAIV, LAIV-G1, and LAIV-G2 viruses. FI-RSV: formalin-inactivated RSV, LAIV: A/PR8 LAIV, including *ts* phenotypic mutations in PB1 and PB2 genes, LAIV-G1: rA/PR8 LAIV-G1 expressing HA-G protein using a linker (GGGGS) between RSV G and HA N-terminal ectodomain, LAIV-G2: rA/PR8 LAIV-G2 expressing HA-G protein using a linker (AAAPGAA) at both G-HA conjugated sites. (**D**) Western blotting of LAIV, LAIV-G1, and LAIV-G2 using mAb 131-2G. 1: Marker, 2: LAIV-G1 (10 µg), 3: LAIV-G2 (10 µg), 4: RSV G-protein (10 µg), and 5: LAIV (10 µg). The arrow indicates chimeric G-HA proteins.

Attenuated A/PR8 backbone (A/PR8 LAIV) was generated by introducing four mutations in the polymerase encoding genes PB1 (K391E, E581G, A661T) and PB2 (N265S) that were known to confer temperature-sensitive (*ts*) and attenuation phenotypes that are similar to those of cold-adapted A/Ann Arbor/6/60 influenza virus, the master donor virus for LAIV [20,21]. Attenuated A/PR8 virus was rescued while using the pHW2000-based eight-plasmid system (kindly provided by Dr. Robert G. Webster, St. Jude Children’s Research Hospital) by means of reverse genetics [17]. The sequence encoding RSV-G central conserved domain (aa 131–230) was cloned between the HA signal peptide and the N-terminal domain of HA1 by using a pHW2000-HA plasmid, as described (Figure 1A) [17]. Two different linker sequences (GGGGS or AAAPGAA) were used between the inserted G-domain and HA and then compared in the antigenic and immunogenic properties of attenuated recombinant A/PR8 viruses (Figure 1A). To rescue attenuated recombinant A/PR8 viruses, 293T cells were transfected with eight pHW2000 plasmids, including PB1 (K391E, E581G, A661T) and PB2 (N265S) mutant genes and chimeric HA-G1 (rA/PR8 LAIV-G1 with GGGGS linker) or HA-G2 (rA/PR8 LAIV-G2 with AAAPGAA linker). At two days culture at 33 °C after transfection of 293T cells, the culture media were collected in order to inoculate into the embryonated chicken eggs. The rescue of recombinant viruses was confirmed by hemagglutination activity assays in egg allantoic fluids collected after 72 h incubation at 33 °C. The recombinant attenuated A/PR8 viruses were then purified by ultracentrifugation and characterized for the expression of HA-G chimeric proteins and attenuated growth properties, as described [17].

### 2.3. Enzyme-Linked Immunosorbent Assay (ELISA) and Western Blot

The expression of chimeric HA-G in attenuated recombinant A/PR8 viruses was determined by the reactivity of rA/PR8 LAIV-G to anti-RSV G specific mAb 131-2G. The rescued viruses purified were coated at different concentrations with ranges 0.125 to 2 µg per well in a 96-well plate. After incubation overnight at 4 °C, the plate was blocked while using 1% bovine serum albumin for 1 h. RSV G-specific mAb 131-2G or A/PR8 virus specific immune sera as primary antibody and anti-mouse IgG-horse radish peroxidase (HRP) as secondary antibody were used in order to determine the reactivity of the recombinant rescued viruses. Western blot was used to detect the chimeric HA-G protein LAIV-RSV G using mAb 131-2G as described [22].

After prime and boost immunization, antigen-specific antibody responses in immune sera were determined by ELISA using RSV G-proteins or inactivated virus as a coating antigen. IgG and IgA antibody levels in lung and bronchoalveolar lavage (BAL) fluid samples at five days after challenge were determined while using ELISA. RSV A2 G-protein fragment (aa131–230) was expressed in *E. coli* and prepared by His-tag affinity column, as previously described [23].

### 2.4. In Vitro and In Vivo Characterization of Recombinant LAIV A/PR8 Viruses Containing Chimeric HA-G

For in vitro growth kinetic tests, Madin–Darby canine kidney epithelial (MDCK) cells were infected with the recombinant A/PR8 viruses. Briefly, 5 × 10^4^ cells per well in 200 µL of complete DMEM (10% FBS, 5% P/S antibiotic) were planted into a 96-well cell culture plate. One day after incubation, the serially diluted recombinant A/PR8 viruses were inoculated into the MDCK culture plate and incubated at 33 °C and 37 °C for five days to determine *ts* phenotypes. The culture supernatants were collected and reacted with 0.5% of chicken red blood cells (RBC, Lampire biological laboratory) in order to determine 50% tissue culture infective dose (TCID_50_) by hemagglutination activity assays.

To determine in vivo attenuated phenotypes, BALB/c mice were infected with recombinant A/PR8 viruses and sacrificed at day 3 after inoculation. Nasal turbinates and lung tissue samples of the mice were collected to determine viral replication in the upper and lower respiratory tracts. The samples were homogenized while using frost glass slides and the extracts were serially diluted to inoculate into 11-day-old embryonated chicken eggs. After three days incubation, the eggs were chilled at 4 °C. The egg allantoic fluids (50 µL) were collected from each egg and then reacted with 0.5% of chicken RBC. EID_50_ (50% egg infective dose) was calculated using a Reed and Muench method [24].

### 2.5. Immunization and RSV Challenge

BALB/c mice (Six to eight-week-old, Jackson Laboratories) were intranasally immunized with 10^6^ EID_50_ (50% egg infectious dose in 50 µL) of attenuated live A/PR8 or chimeric LAIV-G for prime and 5 × 10^6^ EID_50_ (in 50 µL) for boost at a three week-interval. At three weeks after boost immunization, the mice were challenged with 3.5 × 10^5^ plaque forming units (PFU) of RSV A2 strain and then sacrificed at day 5 post infection in order determine lung viral titers and assess histopathology.

### 2.6. Cytokine and Enzyme-Linked Immunospot (ELISpot) Assays

Cytokine levels in the homogenized lung samples and BAL fluids collected at five days after challenge were determined using mouse IFN-γ ELISA ready-set-go kit (eBioscience^TM^, San Diego, CA, USA). The cytokine-secreting cells of splenocytes were determined by ELISpot. Multiscreen 96-well plates (Millipore) were coated with anti-mouse IFN-γ (300 ng/50 µL, BD Pharmingen, San Jose, CA, USA) per well in coating buffer and then incubated at 4 °C. Splenocytes (5 × 10^5^ cells) per well in 100 µL of complete media (RPMI) were planted and stimulated with RSV G-protein (131–230 aa, 4 µg/mL) at 37 °C for 48–72 h. After washing, biotinylated anti-mouse IFN-γ capture antibody was added and incubated for 3 h. Streptavidin-HRP (Southern Biotech, Birmingham, AL, USA) was added after washing in order to develop color using diaminobenzidine (DAB). The spots were counted using ELISpot reader (Bioreader 5000-Eβ, BIOSIS USA).

### 2.7. In Vitro IgG Production as a Measure of Antibody Secreting Cells

The ELISA was performed to measure in vitro production of antibodies from bone marrow (BM), mediastinal lymph node (MLN), and spleen cells. The culture plates were coated with RSV-G (aa131–230) protein [23] or FI- RSV and incubated at 4 °C overnight. After washing, complete RPMI (10% FBS, 1% P/S antibiotics) was added into the plate for blocking and incubated for 1 h. Cells (5 × 10^6^/mL) were seeded into the plates and incubated for one day or five days. The supernatants were collected and added to the G-protein or FI-RSV coated 96-well plates. After 2 h incubation, anti-mouse IgG HRP was added to the plates and incubated for 1 h. TMB was used in order to develop the color and read at 450 nm while using ELISA reader. IgG antibody levels were quantified using standard IgG antibody concentrations under the same ELISA plate.

### 2.8. RSV Plaque Assay for Lung Viral Titration

After sacrificing the mice at day 5 post infection, the extract of the homogenized lung samples was used in order to determine PFU, as described [25]. Serially diluted lung homogenates were added to the Hep2-cell monolayer culture plates. After three days incubation at 37 °C, the plates were fixed with 5% formaldehyde in PBS. The mouse anti-RSV-F mAb and anti-mouse IgG HRP were used to better visualize the immuno-spots using 3,3′-diaminobenzidine tetrahydrochloride substrate (Invitrogen). The lung viral titers were presented by PFU per gram of lung samples.

### 2.9. Histology

The lung tissues were collected and fixed with 10% formalin to analyze lung histopathology. After processing, the tissues were embedded in paraffin and sectioned of 5 µm to stain with hematoxylin and eosin (H&E). Lung histopathology after H&E staining was blind scored in the area of airways, blood vessels, and interstitial spaces with scales of 0 to 3 (0: none, 1: mild, 2: moderate, 3: severe), as detailed in previous studies [26,27,28].

### 2.10. Statistical Analysis

All of the results are expressed as the mean ± standard error of the mean (SEM). The significant differences among treatments were evaluated by one-way or two-way ANOVA, where appropriate. *p*-values of less than or equal to 0.05 were considered to be statistically significant.

## 3. Results

### 3.1. Recombinant A/PR8 Virus with Chimeric HA-G Displays High G Specific Antigenicity

The LAIV A/PR8 HA was engineered in order to conjugate RSV G-conserved domain (aa131–230) into the N-terminus of full-length HA via a GGGGS linker (rA/PR8 LAIV-G1) or AAAPGAA linkers (rA/PR8 LAIV-G2) at both linkage sites (Figure 1A). The recombinant A/PR8 viruses containing chimeric HA-G were generated by a reverse genetic technique while using PB1 and PB2 genes with LAIV phenotypic mutations. The incorporation and expression of chimeric fusion HA-G proteins were determined in recombinant A/PR8 LAIV viruses using RSV G -protein-specific mAb 131-2G and anti-A/PR8 sera by ELISA (Figure 1B,C). rA/PR8 LAIV-G1 and -G2 both showed higher reactivity to G specific mAb 131-2G than FI-RSV, whereas A/PR8 LAIV did not show any reactivity, as expected (Figure 1B). The rA/PR8 LAIV-G1 and -G2 and WT A/PR8 viruses exhibited similar high reactivities to anti-A/PR8 sera in contrast to negative control FI-RSV with no reactivity (Figure 1C), indicating PR8 antigens in LAIV. Furthermore, the expression of chimeric fusion HA-G proteins in rA/PR8 LAIV-G1 and -G2 viruses was evident, as determined by Western blot with RSV-G specific mAb 131-2G (Figure 1D). These results confirm that rA/PR8 LAIV-G1 and -G2 viruses expressing chimeric HA-G proteins display higher RSV G-specific antigenic properties than FI-RSV.

### 3.2. Recombinant A/PR8 LAIV-G1 and LAIV-G2 Viruses Exhibit Limited Replication in the Respiratory Tracts and No Pathogenicity in Mice

**Figure 2:**
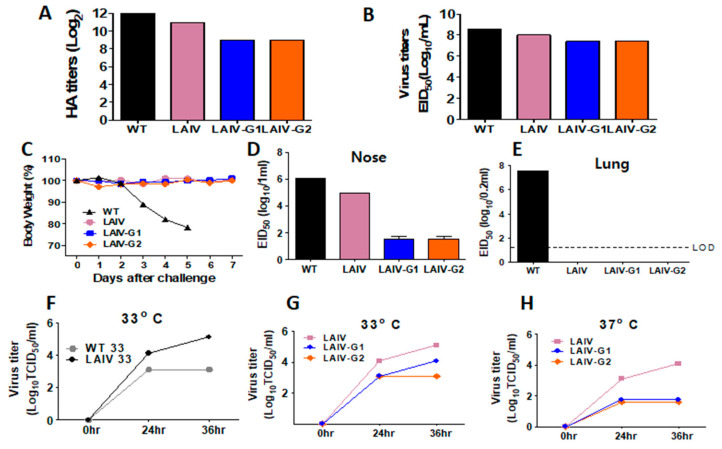
Growth properties and pathogenicity of recombinant A/PR8 LAIV- G1 and LAIV- G2 viruses. (**A**) Hemagglutination activity titers of recombinant rescued LAIV-G1 and LAIV-G2 viruses harvested from egg allantoic fluids. (**B**) Infectious titers in EID_50_ of rescued LAIV-G1 and LAIV -G2 viruses in egg allantoic fluids. (**C**) Pathogenicity in mice. Body weight was monitored after intranasal inoculation of BALB/c mice (*n* = 5) with 10^6^ EID_50_ of LAIV, LAIV-G1, or LAIV-G2. (**D**,**E**) Viral replication titers in the upper (nasal turbinates) and lower respiratory tracts. BALB/c mice were intranasally inoculated with 1 × 10^6^ EID_50_ of LAIV, LAIV-G1, or LAIV-G2. Viral titers were determined in nasal turbinates and lung samples harvested at 3 days after inoculation. (**F**–**H**) In vitro growth kinetics. Serially diluted LAIV, LAIV-G1, or LAIV-G2 viruses were used to infect MDCK cells and incubated at 33 °C and 37 °C. Samples were taken at 0, 24, 36 h post infection in order to determine TCID_50_. LAIV: A/PR8 LAIV including *ts* phenotypic mutations in PB1 and PB2 genes, LAIV-G1: rA/PR8 LAIV-G1 expressing HA-G protein conjugated with a linker (GGGGS), LAIV-G2: rA/PR8 LAIV-G2 expressing HA-G protein conjugated with a linker (AAAPGAA) at both ends. WT 33 and LAIV 33: MDCK cells infected with WT PR8 and LAIV viruses, respectively, and then incubated at 37 °C. WT 37 and LAIV 37: MDCK cells infected with WT PR8 and LAIV viruses, respectively, and incubated at 33 °C.

The growth of the WT A/PR8, A/PR8 LAIV, and rA/PR8 LAIV-G1 and -G2 in eggs was confirmed by HA unit assays. WT A/PR8 and A/PR8 LAIV grew well in eggs showing log of 2^12^ and 2^11^ hemagglutination activity, respectively, as well as high infectious titers of 1~3 × 10^8^ EID_50_ in eggs (Figure 2A,B). The rA/PR8 LAIV-G1 and -G2 viruses also grew well in eggs, displaying 2^9^ hemagglutination activity and ~3 × 10^7^ EID_50_ infectious titers, although their growth levels were moderately lower than those of the control WT A/PR8 and A/PR8 LAIV viruses (Figure 2A,B).

The pathogenic process of influenza A virus begins with infection in the upper airways and leads to replication in the lower respiratory lung tissues. To determine the pathogenicity, the mice were intranasally inoculated with 10^6^ EID_50_ infectious titers of WT A/PR8, A/PR8 LAIV, or rA/PR8 LAIV-G1 and -G2 viruses, and their daily body weight changes were monitored (Figure 2C). All of the mice with A/PR8 LAIV viruses did not show weight loss, whereas the mice with WT A/PR8 virus displayed severe weight loss and died of influenza virus infection. Consistently, mice that were inoculated with WT A/PR8 virus showed the highest level of viral titers in the nasal turbinates and over 10-fold higher viral titers in lungs at day 3 post inoculation when compared to mice with A/PR8 LAIV and rA/PR8 LAIV-G1 or -G2 (Figure 2D,E). The A/PR8 LAIV showed high titers (10^6^ EID_50_) in nasal turbinates, but no infectious titers in lower respiratory lungs (Figure 2D,E), consistent with a typical characteristic of LAIV. The rA/PR8 LAIV-G1 and -G2 viruses showed significantly lower levels of virus replication in nasal turbinates by 10^4^ and 10^3^ folds, respectively, when compared to those of WT A/PR8 and A/PR8 LAIV viruses. (Figure 2D). The replication of the rA/PR8 LAIV-G1 and -G2 was below the limit of detection in lungs (Figure 2E).

In order to determine virus growth kinetics in vitro at low (33 °C) and ambient temperature (37 °C), MDCK cells were infected with WT A/PR8, A/PR8 LAIV, or rA/PR8 LAIV-G1 and -G2 viruses. The A/PR8 LAIV showed faster and higher growth at 33 °C than at 37 °C, which indicated the growth characteristics of influenza viruses with *ts* phenotypes (Figure 2F). Additionally, rA/PR8 LAIV-G1 and -G2 displayed higher growth at 33 °C than at 37 °C, but they showed a lower growth pattern than A/PR8 LAIV at 33 °C (Figure 2G,H). These results suggest that rA/PR8 LAIV-G1 and -G2 viruses are restricted to lower replication in the upper respiratory tract, not replicable in lungs, and not pathogenic to mice, which supports that rA/PR8LAIV-G1 and -G2 viruses display attenuated phenotypes.

### 3.3. Recombinant A/PR8 LAIV-G Virus Vaccines Are Effective in Inducing RSV G-Specific IgG2a Antibodies

**Figure 3:**
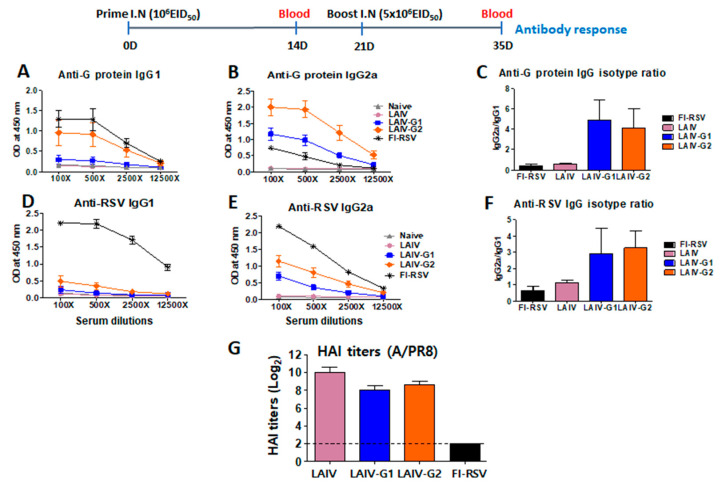
Intranasal inoculation of LAIV-G1 and LAIV-G2 virus effectively induce RSV G specific IgG2a isotype antibody responses. BALB/c mice (*n* = 5) were intranasally (I.N.) immunized with LAIV, LAIV-G1, or LAIV-G2. After prime (1 × 10^6^ EID_50_/100 uL) and boost (5 × 10^6^ EID_50_/100 uL) immunization, sera were collected to measure G-protein and RSV specific IgG1 and IgG2a. (**A**,**B**) G-protein specific IgG1 and IgG2a response after boost immunization. (**C**) Ratios of IgG2a/IgG1 isotype antibodies specific for G protein. (**D**,**E**) RSV specific IgG1 and IgG2a response after boost immunization. (**A**,**B**,**D**,**E**) X axis indicate serum dilutions. (**F**) Ratios of IgG2a/IgG1 isotype antibodies specific for RSV. (**G**) Hemagglutination inhibition (HAI) titers against A/PR8 virus. After prime-boost immunization with LAIV, LAIV-G1, LAIV-G2, or FI-RSV, RSV was challenged, and sera was collected 5 days post infection. HAI titer was measured using the infection sera against A/PR8 virus. FI-RSV: formalin-inactivated RSV (2 µg) with alum adjuvant (50 ug), LAIV: A/PR8 LAIV, including *ts* phenotypic mutations in PB1 and PB2 genes, LAIV-G1: A/PR8 LAIV-G1 expressing HA-G protein using a linker (GGGGS), LAIV-G2: A/PR8 LAIV-G2 expressing HA-G protein using a linker (AAAPGAA).

In order to determine immunogenic responses, BALB/c mice were intranasally immunized with rA/PR8 LAIV-G1 or -G2 viruses in a prime-boost regimen. After prime immunization, the low levels of RSV G-specific IgG antibodies were induced (data not shown). A higher boost dose of LAIV was used to overcome prime immunity against LAIV. However, after boost immunization, notably higher levels of serum RSV G-protein specific IgG2a isotype antibodies were observed in the groups that were immunized with rA/PR8 LAIV-G1 and -G2 than those in the FI-RSV immunized group (Figure 3). Interestingly, the rA/PR8 LAIV-G2 immunization in mice induced higher levels of IgG1 and IgG2a isotype antibody responses than the rA/PR8 LAIV-G1 immunized group (Figure 3A–C). The ratio of IgG2a/IgG1 isotype antibody was significantly higher in rA/PR8 LAIV-G1 and -G2 immunized groups than those in the group with FI-RSV immunization (Figure 3D). Consistently, the higher levels of IgG1 and IgG2a antibody responses that were specific for RSV were induced in the rA/PR8 LAIV-G2 immunized group than those in the rA/PR8 LAIV-G1 immunized group Figure 3D,E). HAI titers against A/PR8 were induced at comparable levels in LAIV, LAIV-G1/G2 groups (Figure 3G). These results suggest that rA/PR8 LAIV-G1 and -G2 vaccines can effectively induce higher levels of RSV G-protein and whole RSV-specific type 1 helper T cells (Th1) IgG2a than type 2 helper T cells (Th2) IgG1 isotype antibodies.

### 3.4. Recombinant A/PR8 LAIV-G Virus Vaccines Provides Protection against RSV Replication

**Figure 4:**
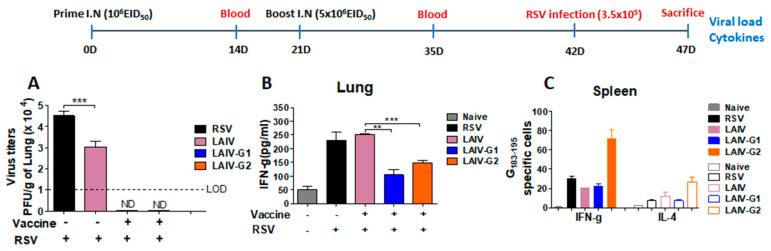
Mice with LAIV-G1 or LAIV-G2 intranasal inoculation are protected against RSV replication. After BALB/c mice were prime (1 × 10^6^ EID_50_/100 µL) and boost (5 × 10^6^ EID_50_/100 µL) immunized intranasally with LAIV, LAIV-G1, or LAIV -G2, mice were challenged with RSV (3.5 × 10^5^ PFU/100 µL) and sacrificed at 5 days post infection to measure lung RSV titers, IFN-γ cytokine levels, and cytokine secreting cells. (**A**) RSV lung viral titers. (**B**) IFN-γ levels in lung. (**C**) IFN-γ and IL-4 cytokine secreting spot-forming cells in spleen as determined by ELISpot. Naïve: no immunization and no infection, LAIV: A/PR8 LAIV including *ts* phenotypic mutations in PB1 and PB2 genes, LAIV-G1: A/PR8 LAIV-G1 expressing HA-G protein using a linker (GGGGS), LAIV-G2: A/PR8 LAIV-G2 expressing HA-G protein using an AAAPGAA linker.: mock control with RSV infection. Statistical significances were performed using one-way ANOVA then Turkey post-test in GraphPad Prism. *** *p* < 0.001, ** *p* < 0.01.

In order to determine protection against RSV challenge, mock control, PR8 LAIV, and rA/PR8 LAIV-G1 and -G2 vaccinated mice were challenged with RSV. The mock control mice showed the highest levels of lung RSV titers (4.5 × 10^4^ PFU/g lung tissue) on day 5 post challenge (Figure 4A). The A/PR8 LAIV group also exhibited significantly higher levels of RSV titers in the lung (3.0 × 10^4^ PFU/g lung tissue) than the rA/PR8 LAIV-G1 and -G2 vaccinated mice. Importantly, the lung viral plaque formation after RSV challenge was below the detection limit in rA/PR8 LAIV-G1 and -G2 vaccinated mice (Figure 4A). These results suggest that rA/PR8 LAIV-G1 and -G2 are effective in controlling RSV replication in the lung of mice after RSV challenge.

The levels of Th1 cytokine IFN-γ were lower in the lungs from rA/PR8 LAIV-G1 or -G2 vaccinated mice than those in mock or A/PR8 LAIV immunized mice after RSV challenge (Figure 4B). However, inflammatory responses are still detectable in these groups, which suggests that RSV might have replicated, although at lower levels. Interestingly, IFN-γ secreting splenocyte cell spots were significantly higher in rA/PR8 LAIV-G2 vaccinated mice than those in other vaccinated groups (Figure 4C). Th2 type cytokine IL-4 secreting spleen cells were relatively lower than IFN-γ secreting splenocytes, although rA/PR8 LAIV-G2 vaccination induced higher G-specific IL-4 secreting cells than other groups (Figure 4C).

### 3.5. Recombinant A/PR8 LAIV-G1 and LAIV-G2 Vaccines Do Not Cause RSV Vaccine-Associated Severe Lung Histopathology

**Figure 5:**
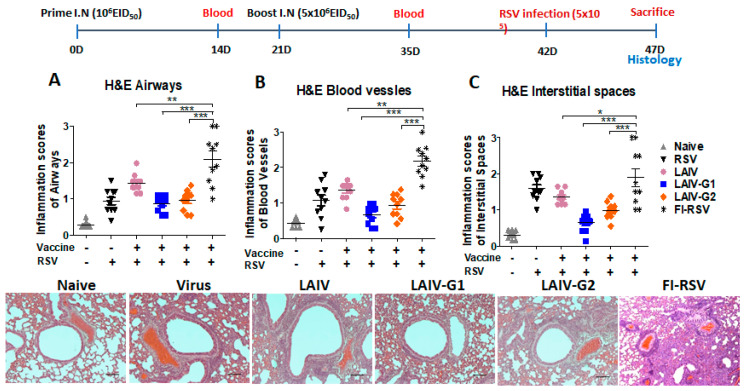
Recombinant PR8 LAIV-G1 and LAIV-G2 virus intranasal vaccination does not cause enhanced lung histopathology upon RSV challenge. After BALB/c mice were prime (1 × 10^6^ EID_50_/100 µL) and boost (5 × 10^6^ EID_50_/100 µL) immunized intranasally with LAIV, LAIV-G1, or LAIV-G2, mice were challenged with RSV (3.5 × 10^5^/PFU/100 µL) and sacrificed at five days post infection to determine lung histopathology (**A**–**C**). Scorings of lung airways, blood vessels, and interstitial spaces were collected from two to three representative histology spots in each mouse after H&E stains. Naïve (gray triangle): mock control without LAIV and RSV, RSV (black triangle): mock control with RSV infection. FI-RSV(black asteric): formalin-inactivated RSV with alum adjuvant, LAIV (pink circle): A/PR8 LAIV including *ts* phenotypic mutations in PB1 and PB2 genes, LAIV-G1 (blue square): A/PR8 LAIV-G1 expressing HA-G protein using a linker (GGGGS), LAIV-G2 (orange diamond): A/PR8 LAIV-G2 expressing HA-G protein using a linker (AAAPGAA), RSV: mock control with RSV infection. *** *p* < 0.001, ** *p* < 0.01, * *p* < 0.05.

FI-RSV immune mice, as a positive control of RSV vaccine-enhanced lung histopathology, on day 5 after RSV challenge displayed severe inflammatory histopathology, as highly scored in the airways, blood vessels, and interstitial spaces (Figure 5A–C). The A/PR8 LAIV control group after RSV challenge exhibited moderate levels of histology inflammation, which is a similar pattern as the mock control, but lower than the FI-RSV group (Figure 5). The rA/PR8 LAIV-G1 and -G2 groups displayed histopathology at low to moderate levels after RSV challenge, as shown in the representative histology and inflammation scores of lung airway and blood vessels as a similar pattern as the mock control, but significantly lower than the FI-RSV group (Figure 5A–C).

### 3.6. Recombinant A/PR8 LAIV-G1 or LAIV-G2 Immunization Induces RSV G Specific Mucosal Antibodies and Antibody Secreting-Cell Responses

**Figure 6:**
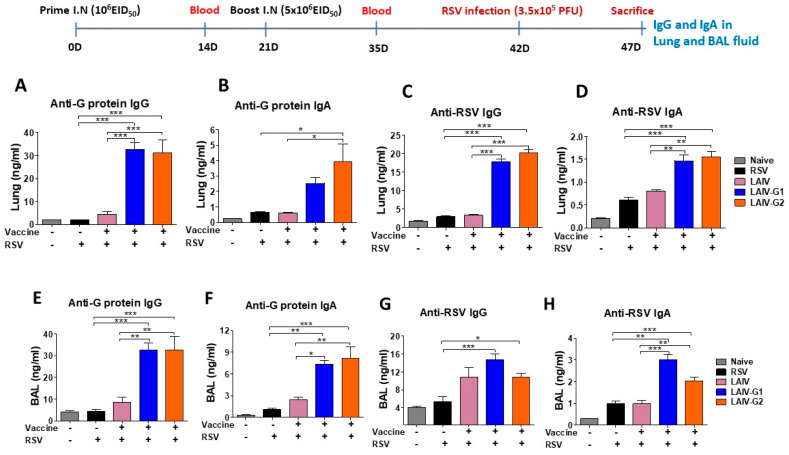
LAIV-G1 and LAIV-G2 virus intranasal vaccination induces RSV G specific IgG and IgA antibody responses in lung and BAL samples. G-protein and RSV specific IgG and IgA antibodies were determined in lung and BAL samples from the mice (*n* = 5) that were immunized intranasally with PR8 LAIV or recombinant PR8 LAIV-G1 or LAIV-G2 at 5 days after RSV infection. (**A**,**B**) G protein specific IgG and IgA in lung. (**C**,**D**) RSV specific IgG and IgA in lung. (**E**,**F**) G protein specific IgG and IgA in BAL. (**G**,**H**) RSV specific IgG and IgA in BAL. Naïve: no immunization and no RSV infection, LAIV: A/PR8 LAIV including *ts* phenotypic mutations in PB1 and PB2 genes, LAIV-G1: A/PR8 LAIV-G1 expressing HA-G protein using a linker (GGGGS), LAIV-G2: A/PR8 LAIV-G2 expressing HA-G protein using a linker (AAAPGAA), RSV: mock control with RSV infection.Statistical significances were performed using one-way ANOVA then Turkey post-test in GraphPad Prism. *** *p* < 0.001, ** *p* < 0.01, * *p* < 0.05.

The levels of mucosal antibodies from lung extracts and BAL fluids were measured at five days after RSV challenge of mice that received intranasal vaccination with rA/PR8 LAIV-G1 and -G2, A/PR8 LAIV virus, or mock. RSV G-protein specific IgG and IgA antibodies induced significantly higher levels in lung and BAL fluid samples from mice with rA/PR8 LAIV-G1 and -G2 immunizations when compared to A/PR8 LAIV and mock control groups (Figure 6A–D). The groups with rA/PR8 LAIV-G1 and -G2 immunizations also induced RSV specific IgG and IgA antibodies in lung and BAL fluid samples at higher levels than the A/PR8 LAIV and mock control groups (Figure 6E–H).

**Figure 7:**
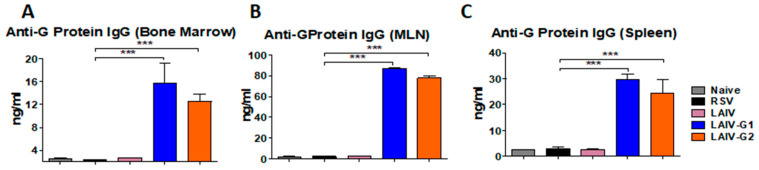

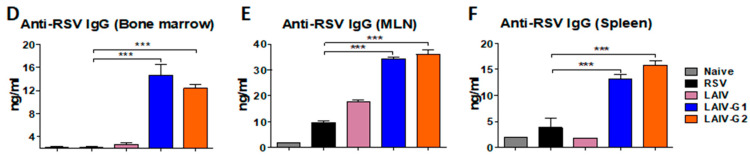
LAIV-G1 and LAIV-G2 virus intranasal vaccination induces RSV G protein specific antibody secreting cell responses in vitro cultures of primary and secondary lymphoid cells. RSV G-protein specific IgG levels were determined in bone marrow (BM), mediastinal lymph node (MLN), and spleen cell culture supernatants from the mice (*n* = 5) that were prime and boost immunized intranasally with LAIV, LAIV-G1, orLAIV-G2 at five days after RSV infection. (**A**–**C**) G protein specific IgG levels in BM, MLN, and spleen cell culture supernatants. (**D**–**F**) RSV specific IgG response in BM, MLN, and spleen cell culture supernatants. Naïve: no immunization and no RSV infection, LAIV: A/PR8 LAIV including *ts* phenotypic mutations in PB1 and PB2 genes, LAIV-G1: A/PR8 LAIV-G1 expressing HA-G protein using a linker (GGGGS), LAIV-G2: A/PR8 LAIV-G2 expressing HA-G protein using a linker (AAAPGAA), RSV: mock control with RSV infection. Statistical significances were performed using one-way ANOVA then Turkey post-test in GraphPad Prism. *** *p* < 0.001.

In order to determine antibody secreting cell responses, BM, MLN, and spleen cells were collected at five days after RSV challenge and prepared for in vitro culture. RSV G-protein specific and whole RSV specific IgG and IgA antibodies were determined in 5 days from in vitro culture supernatants (Figure 7). The BM, MLN, and spleen cells from the rA/PR8 LAIV-G1 and -G2 immunized mice produced G-protein and RSV specific IgG antibodies at significantly higher levels than A/PR8 LAIV and mock controls (Figure 7). The MLN cell culture supernatants produced high levels of RSV specific IgG antibodies (Figure 7), reflecting the generation of plasmablasts that were activated upon RSV infection. Substantial levels of RSV specific IgG antibodies were produced in the spleen and BM cell cultures (Figure 7), supporting the systemic generation of likely long-lived plasma or memory B cells after intranasal vaccination with chimeric HA-G LAIV. The levels of IgG antibodies that were specific for G-proteins were approximately two-fold higher than RSV specific IgG antibodies in MLN and spleen cell cultures. These data suggest that intranasal immunizations of rA/PR8 LAIV-G1 and -G2 effectively induce RSV G-specific mucosal and systemic humoral immune responses possibly with memory B cells, which differentiate to antibody secreting cells.

## 4. Discussion

LAIV is administered in nostrils and it induces mucosal IgG and IgA antibodies by replicating in the upper respiratory track by mimicking natural infection [12]. The G domain with residues aa131-230 inserted in the HA is highly conserved among RSV isolates [29,30]. A previous study reported recombinant WT A/PR8 carrying the RSV G conserved domain, which has intrinsic pathogenic backbone limiting its application as a viral vaccine vector, although protection against RSV was demonstrated [17]. In this study, we generated a recombinant LAIV A/PR8 virus, where its PB1 and PB2 polymerase genes were mutated to mimic LAIV phenotypes. Recombinant A/PR8 LAIV containing HA-G chimeric molecules were highly reactive to RSV G-specific mAb, attenuated, and capable of inducing systemic and mucosal humoral and cellular immune responses, and enabled protection against RSV without causing vaccine-enhanced inflammation. This is the first study demonstrating the use of an attenuated A/PR8 backbone mimicking LAIV in order to express chimeric HA-G proteins conferring protection against RSV infection. The stability of chimeric HA-G LAIV is important as recombinant live vaccine platform or anti-RSV vaccine. LAIV and recombinant chimeric HA-G LAIV showed comparable growth in eggs (Figure 2B). Chimeric molecules of HA-G were stably maintained in recombinant influenza viruses with the wild type backbone A/PR8, even after 10 passages in eggs [17], which suggested that chimeric HA-G LAIV would be quite stable. It will be needed to investigate the stability of chimeric HA-G LAIV in future development. 

Modifications in HA appear to impact on pathogenicity and replication in vivo in mice in addition to polymerase mutations. Backbone A/PR8 LAIV with WT HA was found to exhibit compatible growth capability in eggs, although little less infectious titers (1 × 10^8^ EID_50_ at 33 °C) were observed, as compared to A/PR8 virus with WT backbone (3 × 10^8^ EID_50_ at 37–38 °C). A/PR8 LAIV was highly attenuated as evidenced by *ts* growth in vitro, 10 folds less growth in nasal turbinates, no detection of growth in the lung, and no weight loss in mice after infection, which is consistent with attenuated phenotypes in ferrets, as reported in a previous study [20]. The rA/PR8 LAIV-G1 and -G2 viruses grew well to high titers (2.6–2.9 × 10^7^ EID_50_ at 33 °C), which were slightly less than those that were observed in A/PR8 LAIV with WT HA, in line with an in vitro growth pattern in MDCK cells. In contrast, the growth of rA/PR8 LAIV-G1 and -G2 viruses was highly compromised by a magnitude of 4 log10 lower in nasal virus titers in mice after inoculation, when compared to A/PR8 LAIV with WT HA. The reactivity to RSV G-specific mAb was detected at significantly higher levels in chimeric recombinant A/PR8 LAIV-G1 and LAIV-G2 viruses than whole RSV. This might be due to the high contents of HA being incorporated into influenza viruses up to 30% of total viral proteins [31,32], resulting in high-level incorporation of chimeric HA-G proteins.

The immunogenicity of HA was retained, even with the insertion of the RSV G fragment. The G1 linker was previously used to construct a similar chimeric HA containing a foreign epitope in the N-terminus for rescuing recombinant influenza virus [33]. G2 linker sequences were found to be effective in tandem repeat multi epitope proteins [22]. Therefore, we wanted to compare which linker sequences would be more effective in rescuing recombinant influenza viruses expressing chimeric G-HA proteins and in inducing the immune responses to RSV G fragment inserted. Linkers between the domains in constructing chimeric HA-G constructs appear to play a role in displaying the immunogenic properties of inserted RSV G in HA, although similar antigenic reactivities against RSV G mAb were observed, regardless of the linker sequences. The rA/PR8 LAIV-G2 construct with an AAAPGAA linker was more effective in rescuing chimeric reassortants and inducing G specific IgG2a antibody and IFN-γ cytokine cellular responses than rA/PR/8 LAIV-G1 with a GGGGS linker. It is possible that a flexible linker would enable the foreign antigenic domains in a favorable conformation to be available for the access to B cells or to allow effective intracellular antigen processing and presentation to T cells. The effects of HA insertion sites of foreign antigens and linkers might be prominent in LAIV with limited replication in vivo, since these linker effects were not obvious when expressed in the WT A/PR8 backbone with high replication and pathogenicity in mice [17].

The application of attenuated influenza viruses as a viral vector has advantages. Influenza virus does not involve DNA synthesis during life cycle and, thus, there would be no DNA recombination or integration in host cells, thus increasing the safety aspect. Additionally, the easy manipulation of the antigenic properties of influenza virus using reverse genetics is advantageous in generating recombinant influenza vectored vaccines [14,34,35,36]. The incorporation of foreign RSV neutralizing epitopes in the HA N-terminus in replicating recombinant WT A/PR8 backbone viruses induced humoral immune responses, conferring protection against RSV [16,17]. Recombinant LAIV Leningrad vectors expressing RSV cytotoxic T-cell epitopes inserted into NA and NS1 genes were reported to induce T cell responses conferring protection after prime-boost intranasal inoculation in mice [37,38]. It is likely that viral replication is required for the effective induction of immune responses to a foreign epitope or fragment inserted into the N-terminus of HA, although the efficacy of inactivated HA-G LAIV remains to be determined. LAIV vaccination was reported to exhibit innate immune mediated protective effects on RSV infection [39]. Consistently, we observed reduced lung RSV viral titers by over one log10 magnitude in the LAIV group upon RSV infection (Figure 4A). Therefore, it is possible that the residual innate stimulation via LAIV vaccination promotes the induction of B cell activation and differentiation to plasmablasts in the respiratory draining lymph nodes upon RSV infection (Figure 7E).

## 5. Conclusions

Consistently, this study demonstrates that attenuated recombinant A/PR8 vectors delivering RSV G conserved protective antigenic domain in a chimeric HA, eliciting Th1-biased IgG2a antibody responses in systemic and mucosal sites, confer protection by clearing lung viral loads without vaccine enhanced disease in mice after RSV challenge. Annual influenza vaccination is recommended for all age populations over six months old with inactivated vaccines. Recombinant chimeric influenza vector vaccines might be a dual vaccine candidate providing protection against both influenza virus and RSV. Whereas, LAIV is not recommended for young children less than two years old and the elderly, implicating that recombinant LAIV expressing RSV G would not be appropriate for these high-risk populations. An alternative is to test inactivated recombinant influenza virus vaccines expressing RSV G, a platform that is licensed for young children at six months or older and the elderly. In line with this option, inactivated recombinant influenza virus vaccines expressing RSV fusion protein (F) neutralizing epitopes in an HA chimeric molecule could provide protection against RSV [40], which might be applicable for the elderly. Live attenuated RSV vaccines targeting for young infants are under development [41]. Recurring RSV infections are common and recombinant chimeric HA-G LAIV vaccines might be applicable for boosting RSV G immune responses in children with weak pre-existing RSV immunity.
